# Effects of Advanced Age, Pituitary Pars Intermedia Dysfunction and Insulin Dysregulation on Serum Antioxidant Markers in Horses

**DOI:** 10.3390/antiox9050444

**Published:** 2020-05-21

**Authors:** Agnieszka Żak, Natalia Siwińska, Elżbieta Chełmecka, Barbara Bażanów, Ewa Romuk, Amanda Adams, Artur Niedźwiedź, Dominika Stygar

**Affiliations:** 1 Department of Immunology, Pathophysiology and Veterinary Preventive Medicine, University of Environmental and Life Sciences, 50-375 Wroclaw, Poland; 2 Department of Internal Diseases with Clinic for Horses, Dogs and Cats, Wroclaw University of Environmental and Life Sciences, 50-366 Wroclaw, Poland; natalia.siwinska@upwr.edu.pl (N.S.); artur.niedzwiedz@upwr.edu.pl (A.N.); 3 Department of Statistics, Department of Instrumental Analysis, Faculty of Pharmaceutical Sciences in Sosnowiec Medical University of Silesia, 40-055 Katowice, Poland; ebchelmecka@gmail.com; 4 Department of Pathology, Wroclaw University of Environmental and Life Sciences, 50-375 Wroclaw, Poland; barbara.bazanow@upwr.edu.pl; 5 Department of Biochemistry, Faculty of Medical Sciences in Zabrze, Medical University of Silesia, 41-808 Katowice, Poland; eromuk@gmail.com; 6 Department of Veterinary Science, MH Gluck Equine Research Center, University of Kentucky, Lexington, KY 40546, USA; amanda.adams@uky.edu; 7 Department of Physiology, School of Medicine with the Division of Dentistry in Zabrze, Medical University of Silesia, 41-808 Katowice, Poland; dstygar@sum.edu.pl

**Keywords:** oxidative stress markers, horses, PPID, age, hyperinsulinemia

## Abstract

The study aims to assess the impact of age, pituitary pars intermedia dysfunction (PPID) and insulin dysregulation (ID) in horses on selected oxidative stress markers. The study includes 32 horses, divided into three groups: “young” adult group (aged 8–16 years old) “geriatric” group (aged 18–24 years old) and the “PPID” group (aged 15–31 years old). The PPID group was further divided into two subgroups: PPID ID+ and PPID ID− based on presence or absence of ID. We measured serum antioxidant stress markers in all horses: total oxidant status (TOS), total antioxidant capacity (TAC), ceruloplasmin (CER), lipofuscin (LPS), malondialdehyde (MDA) and thiols concentrations (containing sulfhydryl group -SH) as well as enzymatic systems: total superoxide dismutase (SOD), cytoplasmic SOD (CuZnSOD), mitochondrial SOD activity (MnSOD). Total serum thiols were significantly lower in the geriatric group and in the PPID group compared to the young group. The MnSOD concentration was higher in the PPID ID+ group compared to the PPID ID−. LPS and MDA concentrations were lower in the PPID ID+ group compared to the PPID ID− group. In the selected study groups of horses, older age, the presence of PPID and ID in the case of PPID had no effect on the studied oxidative stress markers.

## 1. Introduction

The mechanism of aging and the development of age-related neurodegenerative diseases in humans and animals are associated with the occurrence of oxidative stress and the action of oxygen free radicals [1,2]. Horses, which are considered long-lived species, are characterized by a high rate of DNA repair and a low rate of free radical production [3,4]. A low rate of free radical production was correlated with a maximum life span in horses when assessed based on the mitochondrial membrane peroxidability index in liver cells [4]. Advanced age can be associated with a weakening of the body’s antioxidant function [5]. Available studies on this subject in horses are ambiguous, showing both a decrease in the activity of superoxide dismutase (SOD) and the total antioxidant capacity with age, as well as a lack of relationship between age and the antioxidant system in horses [6,7]. Advanced age is associated with the occurrence of degenerative changes in the central nervous system both in humans and animals [8]. One example of neurodegenerative disease in horses is pituitary pars intermedia dysfunction (PPID). This disease, formerly called equine Cushing’s disease, primarily affects horses over 15 years of age [9,10]. Age-related changes, as well as oxidative stress, may potentially lead to increased degeneration of the dopaminergic neurons of the hypothalamus [10]. This may result in inhibits of dopamine releasing from the hypothalamus terminal nerve, which results in overproduction of the pituitary gland’s intermediate lobe hormones [10]. These degenerative changes induce cells hyperplasia and formation of micro- and macroadenomas in the pars intermedia, which results in excessive proopiomelanocortin production (POMC) and its derivatives such as adrenocorticotropin hormone (ACTH), α-melanocyte stimulating hormone (α-MSH) and β-endorphin [10]. Typical clinical symptoms include hair coat abnormalities, behavioral disorders, lethargy, chronic laminitis, polyuria, polydipsia, and, in advanced cases, neurological signs [10]. Insulin dysregulation (ID), determined based on confirmation of hyperinsulinemia (HI) or insulin resistance (IR) may occur in the course of PPID [10]. Previous studies describing systemic antioxidant enzymatic systems, such as total glutathione, superoxide dismutase and glutathione peroxidase activity in horses with PPID did not show differences in antioxidant/oxidant equilibrium compared to young, healthy animals [1]. However, in studies on the impact of oxidative disorders on the occurrence of PPID, mitochondrial SOD (MnSOD) activity was found to be locally decreased within the intermediate lobe of the pituitary gland with advanced age in horses [1]. Furthermore, the local activity of 3-nitrotyrosine, a marker of oxidative stress, was increased in both geriatric and PPID horses in the same brain localization [2]. The effect of ID and hyperglycemia on antioxidant status in horses in the course of PPID has only been described in one study that showed an increase in plasma thiols in horses with PPID who have abnormal glucose homeostasis [11].

Based on the current knowledge, we hypothesized that PPID and older age of horses will negatively influence systemic levels of antioxidative stress markers and lead to reduced capacity to combat oxidative stress (OS). Thus, in this study, we aimed to assess serum oxidative status markers in relation to age and neurodegenerative conditions of horses included in the study. A second goal of the study was to assess whether ID is associated with serum antioxidant systems in horses with PPID.

## 2. Materials and Methods

### 2.1. Animals

Thirty-two horses of different sexes and breeds were included in the study. Animals were classified into three groups: the young healthy group (“young”, *n* = 7, age (mean ± SD): 12.1 ± 2.7 years), the geriatric group (“geriatric”, *n* = 6, age (mean ± SD): 22.1 ± 2.2 years) and the PPID group (“PPID”, *n* = 19, age (mean ± SD): 23.6 ± 4.6 years). The PPID group was further divided into the PPID ID+ (PPID horses with HI: *n* = 10, age: 21.7 ± 4.9 years) and PPID ID− (PPID horses without HI: *n* = 9, age: 25.7 ± 3.1 years). All horses underwent a clinical and orthopedic examination, which ruled out abnormalities. All the horses qualified for the study showed no signs or history of laminitis. The horses were included in the groups based on the 2015 Equine Endocrinology Group guidelines for equine PPID diagnosis [12]. During qualification tests: an assessment of the resting ACTH level in venous blood and an assessment of the ACTH level following a thyrotropin–releasing hormone (TRH) stimulation test [12]. This stimulation test was performed by intravenously injecting 1 mg TRH (Sigma-Aldrich, St. Louis, MO, USA) dissolved in sterile saline (1 mg TRH /mL of saline) per horse and measuring the ACTH levels 10 min following TRH administration [12]. Horses were enrolled into the PPID group if: (a) aged > 15 year old, (b) they showed clinical symptoms of PPID, such as hair coat abnormalities, lethargy, polyuria, polydipsia and fat tissue deposition in supraorbital area, (c) their ACTH concentration 10 min after the TRH stimulation test was > 110 pg/mL. Due to a physiological increase in ACTH production by the pituitary gland in horses in the autumn, the study was conducted in March 2017 [13]. All horses were evaluated for resting HI and postprandial HI based on the results of the oral sugar test (OST). The OST was performed using 0.15 mL of corn syrup (Karo, ACH Food Companies, Cordova, TN, USA), per kg of body weight administered orally. Horses were classified in the PPID ID+ subgroup if an insulin concentration after OST was > 45 μU/mL. All the horses were kept under the same environmental conditions at the University of Kentucky’s farm facilities. All the horses were on the same diet, consisting of free choice mixed grass hay and minimal pasture, with access to water and a mineral block ad libitum. The horses remained on the farm for a minimum of two months before the study for acclimatization. All materials and methods were approved by the Institutional Care and Usage Committee of the University of Kentucky (ethical protocol code: nr 2014-1224). The data from some horses were included in another research article about acute phase proteins in horses with PPID; therefore, the clinical assessment and hormone (insulin, ACTH) measurements, are duplicated [14].

### 2.2. Blood Sampling

Blood sampling was performed twice. First, to perform the qualification procedures and second time, 2 weeks after qualifying procedures, for the antioxidants determination (to exclude the effect of performing a TRH stimulation test and OST on the obtained results). Peripheral blood was collected from the jugular vein. Blood serum and blood plasma were isolated.

### 2.3. Hormones

Insulin concentration was measured with a commercially available human insulin radioimmunoassay (RIA) (EMD Millipore Corp, Billerica, MA, USA) that was validated for use in equine serum samples, as previously described [15]. ACTH was measured with a previously validated chemiluminescent assay (Immulite, Diagnostics Product Corporation; Los Angeles, CA, USA) [15].

### 2.4. Protein Concentration

The assessment of protein concentration in the selected samples was performed according to the Lowry method [16].

### 2.5. Antioxidants

Antioxidant systems in serum samples were analyzed by assessing the enzymatic activity of total superoxide dismutase (SOD, EC 1.15.1.1) and non-enzymatic activity was assessed by measuring the concentration of lipofuscin (LPS), the concentration of ceruloplasmin (CER), serum thiols concentrations (SH), total oxidant status (TOS) and total antioxidant capacity (TAC). The level of lipid peroxidation was assessed by measurement of serum malondialdehyde (MDA) concentration.

#### 2.5.1. Superoxide Dismutase (Total SOD, CuZnSOD, MnSOD)

The Oyanagui method was applied to measure the activity of SOD isoenzymes [17]. In that method, KCN is used as the inhibitor of the CuZnSOD isoenzyme. The activity of CuZnSOD was evaluated as the difference between total SOD activity and MnSOD activity. The activity of the SOD isoenzymes was presented as nitrite units (NU) per mL serum. One NU exhibits 50% inhibition of the formation of nitrite ion under the method’s condition.

#### 2.5.2. Lipofuscin (LPS)

Serum levels of LPS were assessed using Tsuchida et al. methodology [18]. Serum samples of selected horse were diluted with 3:1 *v/v* ethanol–ether, shaken and centrifuged. The intensity of fluorescence was determined at a wavelength of 345 nm for absorbance and 430 nm for emission in a dissolved solid. The results are presented as relative lipid extract fluorescence.

#### 2.5.3. Ceruloplasmin (CER)

Serum ceruloplasmin concentration was measured according to the Richterich method [19]. In that method, ceruloplasmin catalyzes the oxidation of *p*-phenylenediamine. The rate of formation of the colored product is proportional to the ceruloplasmin concentration and may be directly determined by spectrophotometry. The absorbance of the samples was read at 560 nm and the results are expressed as mg/mL.

#### 2.5.4. Total Oxidant Status (TOS)

Plasma samples were assayed for TOS with the commercially available total oxidant status (TOS) kit (Randox Laboratories, Ltd., Crumlin, England) using Trolox as a standard (6-Hydroxy-2,5,7,8-tetramethylchroman-2-carboxylic acid). The assay results are expressed as a Trolox equivalent (mmol/L).

#### 2.5.5. Total Antioxidant Capacity (TAC)

The assessment of TAC was performed according to the Erel methodology [20]. This method uses the oxidation of ferrous iron ions to ferric iron ions in an acidic medium. The range of formation of a colorful product blue-purple in color was read at a 560 nm. The TAC values are expressed in μmol/L.

#### 2.5.6. Lipid Peroxidation

The serum malondialdehyde (MDA) concentration was determined using the reaction with thiobarbituric acid (TBA) followed by the condensation of two molecules of TBA with one molecule of MDA and the elimination of two molecules of water to obtain a TBA pigment, measured spectrophotometrically. The MDA concentration was calculated from the standard curve [21].

#### 2.5.7. Serum Thiols (Containing Sulfhydryl Group -SH)

The serum concentration of sulfhydryl groups (-SH) was determined according to Koster et al., using a semi-automatic method and a VICTOR-X3 reader (PerkinElmer, Inc., Waltham, MA, USA) with a 405 nm filter [22]. The method is based on the use of 5,5′-dithiobis (2-nitrobenzoic acid) (DTNB), which is reduced by compounds containing sulfhydryl groups. The product of this reaction is a yellow 5-thio-2-nitrobenzoic anionic derivative. The concentration of sulfhydryl groups was calculated from the calibration curve using reduced glutathione as a standard. The basis for the calculations was the absorbance difference of the test sample after subtracting the corresponding blank value.

### 2.6. Statistical Analysis

Statistical analysis was performed using STATISTICA 13 PL (TIBCO Software Inc. (2017), Statistica (data analysis software system), version 13). Interval data were expressed as mean values ± standard deviation in the case of a normal distribution or as median / lower–upper quartile range [Me (Q1–Q3)] in the case of data with skewed or non-normal distribution. The distribution of variables was evaluated using the Shapiro–Wilk test and the quantile–quantile plot. The Student’s—*t*-test or ANOVA with the post hoc Tukey’s test were used to compare data. If the data were skewed, logarithmic transformation was performed before their analysis. A 95% confidence interval (CI) was specified. Statistical significance was set at a *p*-value below 0.05. All the tests were two-tailed.

## 3. Results

The inter- and intra-assay coefficients of variation (CV) for: SOD were 2.8% and 5.4%, respectively; for LPS measuring were 2.8% and 9.7%, respectively; for CER measuring were, respectively, 1.3% and 4.0%; for TOS measuring were 1.1% and 3.8%, respectively; for TAC measuring were 2.1% and 4.2%, respectively; for MDA measuring were 2.1% and 8.3%, respectively; for -SH measuring were 2.6% and 5.4%, respectively.

The results of endocrine tests (resting ACTH, TRH stimulation test, basal insulin concentration, OST) and the detailed information on study groups (number of horses, age) are presented in Table 1. In the young group and the geriatric group, the concentrations of resting ACTH, ACTH after TRH stimulation test, resting insulin and insulin after OST were within reference intervals.

**Figure 1:**
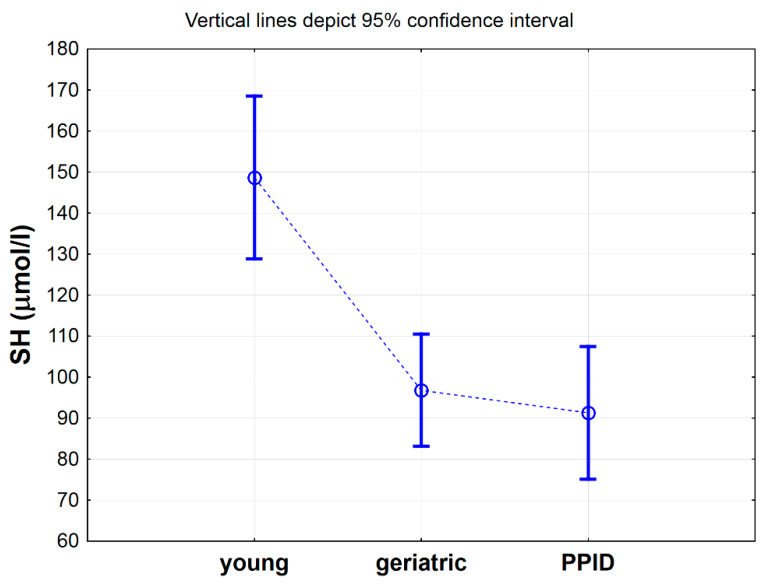
Mean values of total thiol -SH concentration measured in serum of horses in young, geriatric and pituitary pars intermedia dysfunction (PPID) group. Statistical significance was set at *p* < 0.05. The statistical significance difference between young group vs. geriatric and PPID groups was shown.

Table 2 shows a comparison of selected antioxidative markers between control, geriatric and PPID groups of horses. Statistical analysis showed significant differences only for -SH (*p* < 0.001), Total SOD (*p* < 0.01), CuZnSOD (*p* < 0.05), CER (*p* < 0.05) and MDA (*p* < 0.05) between analyzed groups (Table 2). Nevertheless, the results of post hoc analysis showed significant differences only for -SH groups concentrations [µmol/L], which were significantly higher in the control group when compared to geriatric group (*p* < 0.01) and PPID group (*p* < 0.01) (Table 2, Figure 1). When the concentration of the -SH was albumin-corrected, the values were 2.1 µmol/g albumin (SD = 0.2) in the control group, 1.4 µmol/g albumin (SD = 0.2) in the geriatric group (*p* < 0.05) and 1.3 µmol/g albumin (SD = 0.5) in the PPID group (*p* < 0.01) and were lower than those previously determined in cited publication for healthy horses (8.4–9.6 µmol/L/g albumin) [23].

**Figure 2:**
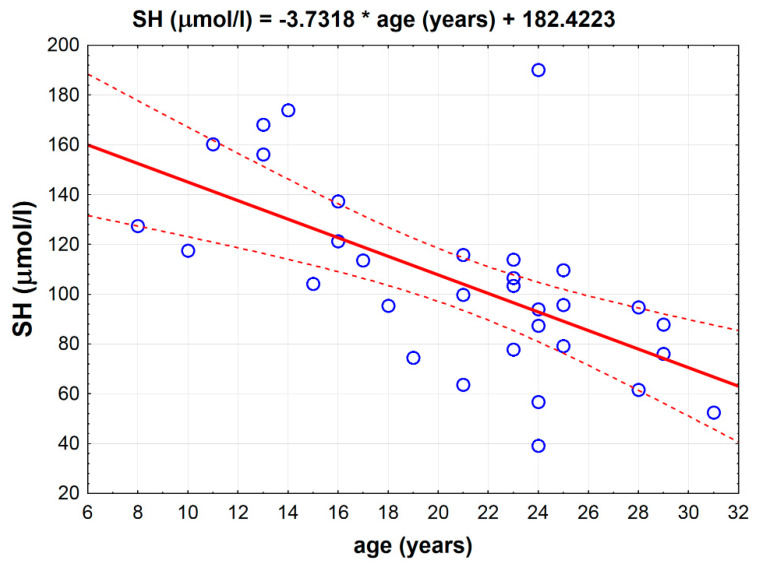
Inverse correlation between total serum thiol -SH concentration and age in all horses (*p* < 0.001, *r* = −0.6143).

There was a strong inverse correlation between age and the total thiol -SH concentration measured in the serum (*p* < 0.001, *r* = −0.6143) independently from the groups (Figure 2).

**Figure 3:**
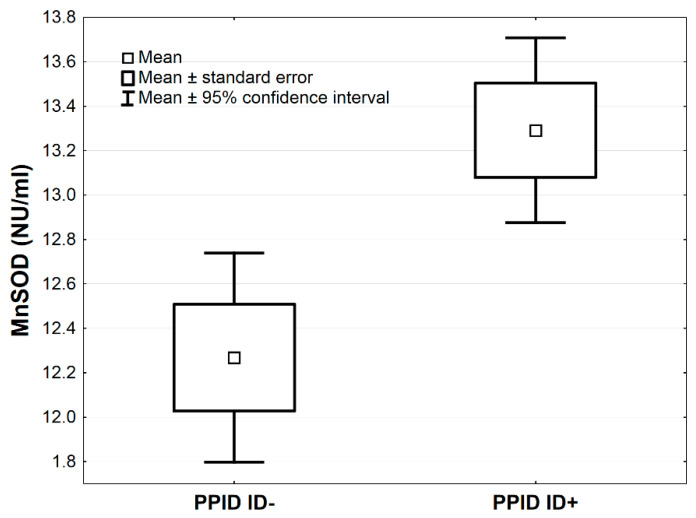
Mean values of MnSOD activity measured in serum of horses in PPID ID+ and PPID ID− groups. Statistical significance was set at *p* < 0.01.

**Figure 4:**
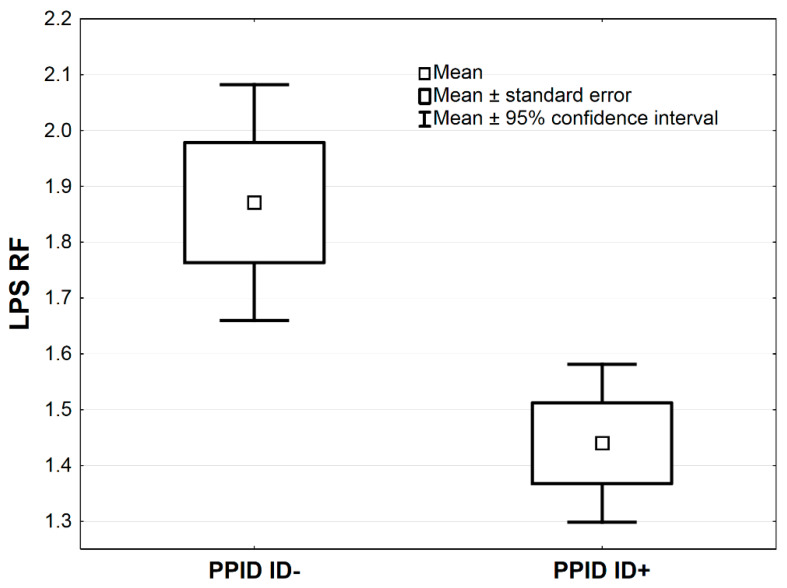
Mean values of LPS activity measured in serum of horses in PPID ID+ and PPID ID− group. Statistical significance was set at *p* < 0.01.

**Figure 5:**
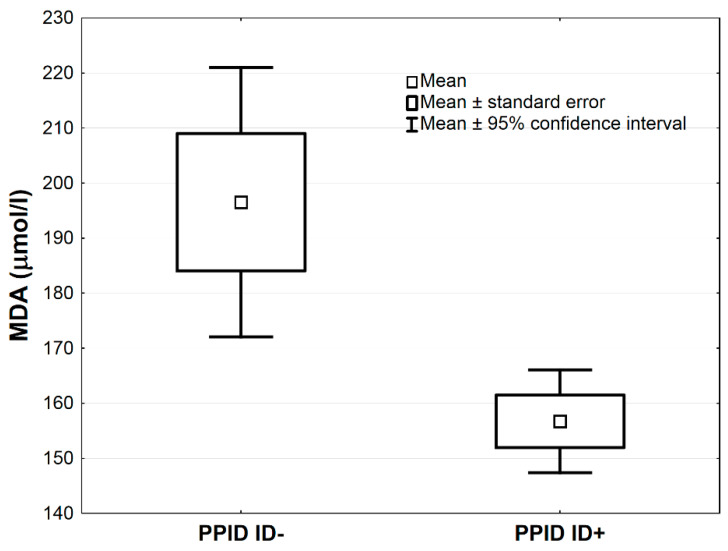
Mean values of serum malondialdehyde (MDA) activity in PPID ID+ and PPID ID− group. Statistical significance was set at *p* < 0.05.

In Table 3, the comparisons of antioxidative markers between PPID ID+ vs. PPID ID− groups are presented. The MnSOD concentration was higher in the PPID ID+ group compared to the PPID ID− group by the average 1.0 NU/mL, 95% CI: (0.3–1.7), (*p* < 0.01, Table 3; Figure 3). The LPS and MDA concentrations were lower in the PPID ID+ group compared to the PPID ID− group by an average 0.4, 95% CI: (0.2–0.7) and an average 39.8 µmol/L, 95% CI: (12.8–66.9), respectively (*p* < 0.01 and *p* < 0.05, respectively; Table 3, Figure 4 and Figure 5). No other significant differences were observed between the PPID ID+ and PPID ID− subgroups.

## 4. Discussion

In horses, so far, oxidative stress was extensively described under pathological conditions such as non-infectious respiratory diseases: severe asthma and inflammatory airways disease, but not in case of endocrine disorders [24,25]. Changes in the oxidant/antioxidant equilibrium have also been observed in case of neurological disorders such as equine grass sickness [23,26], equine motor neuron disease [27]; myopathy known as “tying up syndrome” [28] and also in cases of joint diseases [29]. A number of studies have investigated the antioxidant/oxidant equilibrium associated with high-speed and long-distance physical activity during training. Physical effort, as well as physical exercise, have a significant positive impact on the antioxidation ability in horses [30,31]. The occurrence of oxidative stress and the functioning of the antioxidant systems in the cells are dependent on many external factors other than age and disease. Factors that influence antioxidant status include, above all, environmental factors, diet and the use of the animals [6,7,32]. An increase in oxidative stress may be associated with long-term transport [33] or contamination of the environment with heavy metals and fertilizers [34]. The oxidant/antioxidant equilibrium in horses may also be dependent on breed and gender [35]. In the present study, we selected the groups in such a way as to eliminate the influence of environmental factors, maintenance and diet on the results. However, the examined horses were mixed breed and of both sexes which made it impossible to eliminate the impact of these parameters. In this study, we have, for the first time, shown that (i) older age is negatively correlated with serum thiols concentrations in horses; (ii) the presence of PPID did not change the status of antioxidative stress markers significantly; (iii) the presence of ID in PPID horses does not detectably influence the cellular defense against ROS.

In horses, the relation between age and the antioxidative profile was partially examined. To date, the studies assessing the effect of age on antioxidant abilities were carried out on young horses [6,7,31]. Just two studies: of Williams et al. 2008 and Mochizuki et al. 2016 were performed on horses on average 22 years and from 35 years old and showed no effect of age on the diacron-reactive oxygen metabolites or the biologic antioxidant potentials [30,36]. The only available data assessing the occurrence of a local oxidative stress reaction associated with age in horses were assessed based on negative correlation between the MnSOD concentration in the intermediate lobe of the pituitary gland and age [1]. The described results suggested that oxidative stress was a cause of neurodegenerative changes in the pituitary gland in horses [1]. A reduction in the total antioxidant activity in older humans compared to a young control group was shown [5]. The MDA:TAC ratio was considered a significant marker of oxidative stress, which increases significantly with age, suggesting a decrease in the overall antioxidant capacity [37]. Based on the results of this study, there was no increase in MDA:TAC ratio in elderly horses.

In our study, we report a significant decrease in the concentration of serum thiols with age and PPID (i.e., in horses between 15 and 31 years old) when compared to the control group (horses aged between 8 and 16 years old). In addition, a strong negative correlation was observed between serum thiol concentrations and age. The obtained results are in line with the data obtained in human subjects, which showed both a negative correlation of the concentration of thiols with age as well as a decrease in plasma thiol concentration in the geriatric group compared to the young control group [38,39]. Other studies confirm that plasma thiols decrease in horses with PPID; however, it was not determined whether this decrease was associated with age [11]. Thiols are sensitive and specific indicators of systemic oxidative stress and their biologic activity depends on free radical detoxification and the protection of cells against electrophilic xenobiotics [40]. Their decrease with age in horses may be associated with a decrease in the body’s antioxidant capacities. However, in case of our study, the lack of differences in the concentration of MDA, TOS, TAC and enzymatic antioxidant concentrations suggests sufficient compensation for oxidative stress by the antioxidant system in older and PPID horses. The presence of differences in the concentration of thiol groups per g of protein in the horses in the present study compared to those reported by McGorum et al. 2003 is interesting, as the same methodology to assess thiol concentrations was applied in both studies [23]. This difference may also result from various environmental factors, the amount of ingested antioxidants and the type of horse used.

PPID is a neurodegenerative disease affecting dopaminergic neurons of the hypothalamus and the secretory function of the pituitary pars intermedia [10]. Oxidative stress is recognized as one of the potential causes of neurodegenerative changes in this disease, in addition to age-related differences or environmental factors [10]. In horses with PPID, there is a 16-fold increase in the levels of 3-nitrotyrosine, an oxidative stress marker, in the nerve terminals of the periventricular dopaminergic neurons compared with healthy adult horses [2]. In PPID horses, there were an increase in the number of lipofuscin pigments within the pituitary nerves [41]. A mild decrease of plasma thiols in horses with PPID [11] or a lack of changes in the systemic antioxidant profile were also reported [1]. The present studies showed a decrease in serum thiols in PPID geriatric horses compared to the non-PPID control group of younger horses. However, this decline was also associated with advanced age and not just PPID. Additionally, no antioxidant failure in the horses with PPID was found in this study, which confirmed the available data in the literature. There was no correlation between the ACTH concentration and the measured parameters. Based on current knowledge of the pathomechanism of PPID, further research are required to determine the occurrence of local oxidative stress within the hypothalamic dopaminergic nerve terminals, as they undergo degenerative changes. Studies performed in humans focus on clarifying whether oxidative stress is a cause or effect of degenerative changes [42]. Due to the similarity in the pathogenesis of PPID in horses and spontaneous dopaminergic neurodegeneration that occurs in Parkinson’s Disease (PD) in human subjects, the former is considered an animal model for the latter [43]. The results reported from human studies on PD showed a decrease in TOS, total SOD and glutathione peroxidase (GPx) concentrations compared to healthy controls [44]. It was shown that total SOD activity decreases with the severity of PD in human subjects [44]. Results obtained in our study may indicate, that older horses and horses with neurodegenerative disorders show compensatory abilities; and their antioxidant system may be more efficient in neutralizing oxidative stress than in people.

In the course of PPID, horses may develop ID marked by resting or postprandial HI as well as IR [10]. ID may be related to the presence of laminitis in horses with PPID, as well as the increase in oxidative stress, which is considered a potential cause of PPID [10,45,46]. However, there is no in-depth analysis of the cause–effect mechanism between these disorders in the literature. Dysregulation in glucose metabolism within the capillaries of the hooves is correlated with a local increase in ROS production in horses [47]. A comparison of ponies that had a history of laminitis with healthy ones showed no changes in markers of antioxidant function and oxidative pressure, despite the presence of IR in the studied group [48]. However, there is a lack of complete data that would indicate the effect of hyperinsulinemia on the antioxidant profile, not related to the presence of laminitis in horses. Insulin dysregulation is known to be associated with lower concentrations of trans-4-hydroxyproline and methionine sulfoxide, which may indicate that oxidative stress and oxidant/antioxidant disequilibrium are contributing factors to insulin dysregulation in horses [49]. However, there are no large-scale studies comparing the effect of ID on the antioxidant profile [49]. In the study by Keen et al. HI was observed in some horses from the PPID group (termed ECD), but no individual differences in the antioxidant profile that could have resulted from ID in the course of PPID were found [49]. In humans, metabolic syndrome is associated with increased oxidative stress, which has been suggested to be a component in the development of IR [50]. The present study showed significant differences in the activity of three antioxidant enzymes depending on the occurrence of ID. There were no differences in the total SOD between the PPID ID+ and PPID ID− subgroups, although the mitochondrial component (MnSOD) was significantly higher in the PPID ID+ subgroup. MnSOD plays a role as an antiapoptotic factor, acts against ROS and reduces the effects of inflammatory reagents [51]. Under conditions of insulin deregulation, the concentration of MnSOD was significantly increased, which may be understood as intensification of compensatory processes in order to clear the mitochondrial ROS and thus protect them against cell death. The results obtained from MnSOD transgenic mice showed that mitochondrial superoxide protected from diet-induced oxidative stress as well as enhanced the control of insulin acting as a metabolic sensor of energy excess [52]. Oxidative stress markers, i.e., LPS and MDA concentrations, were lower in the subgroup with ID, compared to the PPID ID− subgroup. Based on these results, we can conclude that the occurrence of ID in the setting of PPID does not stimulate an increase of oxidative stress in the selected group of horses. This differences in LPS and MDA concentrations may be explained by a greater age of the horses in the PPID ID subgroup, compared to the PPID ID+ group. Nevertheless, this hypothesis must be supported by further analysis of the antioxidant systems in horses with PPID and ID.

In our work, we did not observe significant differences in the level of lipid peroxidation measured by MDA concentration between selected study groups. In addition, TAC and other antioxidant stress markers (except -SH) were similar in young, geriatric and PPID groups. Primarily, this may suggest that PPID and age of selected horses were not strong inducers of OS and/or antioxidant mechanisms were sufficient to reduce mild OS caused by those two selected factors. Lack of significant differences between study groups may be also an effect of relatively low number of samples included in the study, which we consider a main limitation of the present work.

## 5. Conclusions

We can conclude that in the selected study groups, older age and the presence of PPID did not change the status of antioxidative stress markers significantly. Similarly, the presence of ID in PPID horses did not considerably influence the cellular defense against ROS.

## Figures and Tables

**Table 1:** Detailed information on study groups and the results of endocrine tests.

Group	*n*	Age (Mean ± SD)	ACTH T0 [pg/mL] (Median, Q1–Q3)	ACTH T10 [pg/mL] (Median, Q1–Q3)	Insulin T0 [µU/mL] (Median, Q1–Q3)	Insulin T60 [µU/mL] (Median, Q1–Q3)
Young	7	12.1 ± 2.7	19.5 (18.1–21.1)	32.9 (27.3–44.1)	16.9 (11.3–23.9)	40.9 (21.5–41.9)
Geriatric	6	22.0 ± 2.2	26.0 (18.2–34.1)	49.3 (35.6–57.9)	21.5 (17.2–26.0)	28.8 (22.8–39.4)
PPID	19	23.6 ± 4.6	51.4 (25.3–91.3)	524.0 (129.0–829.0)	22.7 (19.8–52.4)	56.2 (21.5–129.5)
PPID ID+	10	21.7 ± 4.9	38.4 (19.9–59.9)	416.0 (166.0–707.0)	49.6 (28.6–86.9)	120.2 (66.2–149.1)
PPID ID−	9	25.7 ± 3.1	77.4 (46.2–188.0)	631.0 (129.0–1195.0)	19.8 (15.4–22.3)	21.5 (19.6–39.2)

ACTH T10: adrenocorticotropin hormone (ACTH) post thyrotropin–releasing hormone (TRH) stimulation test; insulin T60: insulin post oral sugar test (OST); Q1: first quartile is defined as the value that has an *f*-value equal to 0.25; Q3: The third quartile has an *f*-value equal to 0.75.

**Table 2:** Comparison of selected antioxidative markers between control, geriatric and PPID groups.

Antioxidative Markers	Young *n* = 7	Geriatric *n* = 6	PPID *n* =19	*p*_(ANOVA)_	*p* Young vs. Geriatric	*p* Young vs. PPID	*p* Geriatric vs. PPID
**Protein (g/L)**	71.0 ± 4.4	66.7 ± 2.7	68.9 ±3.9	0.146	–	–	–
**SH (µmol/L)**	148.7 ± 21.5	96.8 ± 13.0	91.3 ± 33.5	**<0.001**	**<0.01**	<0.01	0.940
**CER (mg/dL)**	17.0 ± 4.1	16.6 ± 3.9	13.9 ± 2.9	**<0.05**	0.970	0.163	0.303
**TAC (mmol/L)**	0.67 ± 0.05	0.63 ± 0.04	0.63 ± 0.04	0.102	–	–	–
**TOS (µmol/L)**	3.8 ± 2.2	4.3 ± 1.7	7.2 ± 4.1	0.055	–	–	–
**Total SOD (NU/mL)**	20.1 ±1.2	19.8 ± 1.0	21.1 ± 1.1	**<0.05**	0.896	0.208	0.117
**MnSOD (NU/mL)**	13.3 ± 1.1	12.3 ± 1.3	12.8 ± 0.9	0.217	–	–	–
**CuZnSOD (NU/mL)**	6.8 ± 1.7	7.5 ± 0.5	8.3 ± 1.4	**<0.05**	0.632	0.111	0.579
**LPS RF**	1.6 ± 0.3	1.5 ± 0.3	1.6 ± 0.3	0.714	–	–	–
**MDA (µmol/L)**	203.2 ± 46.8	215.3 ± 31.1	175.6 ± 34.0	**<0.05**	0.833	0.395	0.157

PPID: pituitary pars intermedia dysfunction; SH: serum thiols; CER: ceruloplasmin; TAC: total antioxidant capacity; TOS: total oxidant status; SOD: superoxide dismutase; MnSOD: mitochondrial SOD; CuZnSOD: cytoplasmic SOD; LPS RF: lipofuscin relative fluorescence; MDA: malondialdehyde.

**Table 3:** The comparisons of antioxidative markers between PPID ID+ vs. PPID ID− groups.

Antioxidative Markers	PPID ID− *n* = 9	PPID ID+ *n*=10	*t*	*p*
**Protein (g/L)**	69.1 ± 4.7	68.8 ± 4.7	0.197	0.846
**SH (µmol/L)**	78.9 ± 21.3	102.4 ± 39.4	1.585	0.131
**CER (mg/dL)**	14.3 ± 3.0	13.1 ± 2.7	0.944	0.359
**TAC (mmol/L)**	0.63 ± 0.03	0.64 ± 0.04	0.806	0.431
**TOS (µmol/L)**	6.0 ± 2.6	7.0 ± 3.4	0.691	0.499
**SOD (NU/mL)**	21.1 ± 1.2	21.1 ± 1.0	0.104	0.918
**MnSOD (NU/mL)**	12.3 ± 0.7	13.3 ± 0.7	3.205	**<0.01**
**CuZnSOD (NU/mL)**	8.8 ± 1.1	7.7 ± 1.5	1.826	0.086
**LPS RF**	1.9 ± 0.3	1.4 ± 0.2	3.382	**<0.01**
**MDA (µmol/L)**	196.5 ± 37.5	156.7 ± 15.1	2.981	**<0.05**

PPID ID+: Group of horses with Pituitary pars intermedia dysfunction and insulin dysregulation; PPID ID−: Group of horses with Pituitary pars intermedia dysfunction without insulin dysregulation.
